# Extraction of Needles after Location with the *X*-Rays

**Published:** 1904-03-26

**Authors:** 


### EXTRACTION OF NEEDLES AFTER LOCATION WITH THE X-RAYS.

Everyone who has attempted the extraction of portions of needles and similar foreign bodies which have become buried in the tissues, will know how frequently the seemingly simple operation ends in complete failure, or is followed by troublesome and prolonged suppuration. A useful hint is given by E. W. Shenton[1] for expressing the foreign body after locating it with the *x*-rays and fluorescent screen. The following is his method, which, seeing that it has been successfully employed at Guy's Hospital in some hundreds of cases, can certainly be said to have had a fair trial. As soon as the needle is clearly seen on the screen, the part is manipulated until the needle shadow is perfectly foreshortened so that it is represented by a mere dot. A mark is then made on the skin exactly over the shadow of the needle both on the front and back surface of the hand or whatever other part is being treated. It is clear that the long axis of the needle will coincide with the line joining the two marks on the skin. The *x*-ray apparatus is no longer needed and the current is turned off. The operator's forefinger and thumb are then placed exactly over the dots, and approximated. Pressure is then released for a moment and applied again. It will be found, Mr. Shenton states, that every time pressure is correctly applied the needle moves in a constant direction, namely that to which its finer end is pointing. If the actual point is on the imbedded fragment the process is rapid, but even if a portion of the shaft only of the needle is present the method will succeed provided that one end be narrower than the other, which will usually be the case. Sometimes after the first squeeze, at others after much manipulation, one end of the fragment may be felt immediately under the skin; and if the end is too blunt to be thrust through the skin, a small nick with the point of a scalpel is all that is necessary to allow the foreign body to escape. If the pressure is made slowly, but little pain is caused to the patient. Thus in many cases the needle can be removed without the use of a general anæsthetic and without the fear of causing suppuration, while should the plan fail it is always possible to resort to an open incision.

Mr. Shenton remarks that this method is only an adaptation of the natural process by means of which needles will travel in the soft tissues. He mentions an interesting example of this migratory capacity of foreign bodies. A girl complained of having run a needle into her neck when laying her head upon a pillow. Upon examination there was a small skin puncture just below and behind the mastoid process. The *x*-rays showed the needle lying beneath the puncture, and on deep pressure the end could be felt. Owing to some mistake the girl went home and was not immediately operated on. Two days later she returned to hospital, when an incision was made but no needle found. Eight days after this, by means of the $x$-rays, the foreign body was found near the superior angle of the scapular, and extracted. It is clear that in this case the needle travelled nearly the whole length of the neck in the space of 10 days.

[1] Med. Elect. and Radiol., Feb. 1904.

